# Supplementary material for: MetaRibo-Seq measures translation in microbiomes
Source: Nat Commun. 2020 Jun 29;11:3268. doi: 10.1038/s41467-020-17081-z (PMC7324362; doi:10.1038/s41467-020-17081-z)
Supplement: Supplementary file 10 — Supplementary Data 7 [file 41467_2020_17081_MOESM10_ESM.zip › File2/Confidence_High_Taxonomy/133640_out.krona.html]

Javascript must be enabled to view this page.

members
magnitude
magnitudeUnassigned
count
unassigned
taxon
rank

133640\_out

6

superkingdom
6
2

6
1239
phylum

186801
6
class

186802
6
order


SRS013476\_contig\_number\_contig-100\_202.190312SRS024132\_contig\_number\_44874SRS078419\_contig\_number\_contig-100\_3772.186410SRS144135\_contig\_number\_26553SRS146812\_contig\_number\_57415SRS150029\_contig\_number\_20495
1898207
6
species
